# Transcriptome and gene co-expression network analysis revealed a putative regulatory mechanism of low nitrogen response in rice seedlings

**DOI:** 10.3389/fpls.2025.1547897

**Published:** 2025-06-10

**Authors:** Bright G. Adu, Yoshihiro Ohmori, Astushi J. Nagano, Toru Fujiwara

**Affiliations:** ^1^ Department of Applied Biological Chemistry, Graduate School of Agricultural and Life Sciences, The University of Tokyo, Tokyo, Japan; ^2^ Agricultural Bioinformatics Research Unit, Graduate School of Agricultural and Life Sciences, The University of Tokyo, Tokyo, Japan; ^3^ Faculty of Agriculture, Ryukoku University, Otsu, Japan; ^4^ Institute of Advanced Biosciences, Keio University, Tsuruoka, Japan

**Keywords:** low nitrogen tolerance, wild rice introgression lines, transcriptome, gene coexpression, cell wall biogenesis, ion transport

## Abstract

In rice, nitrate (NO_3_^−^) and ammonium (NH_4_^+^) are the main sources of inorganic nitrogen (N) for growth, which also serve as signaling molecules. Depending on the N status, plants modulate their physiological traits such as root system architecture (RSA) and transcriptome makeup, including N uptake and assimilation genes, to adapt to the amount of N available in the growth medium. In this study, time-course hydroponic experiment under low N (0.4 mM NH_4_^+^) and sufficient N (1.6 mM NH_4_^+^) was performed using low N tolerant introgression lines, KRIL8 and KRIL37, which carry a small region of the wild rice *Oryza rufipogon* genome in the *Oryza sativa* L. cv Koshihikari background. RNA-Seq analysis was used to profile changes in gene expression related to N and carbon metabolism which varied significantly and identified the accumulation of transcripts involved in secondary metabolite synthesis at the peak of low N stress. Weighted gene co-expression network analysis (WGCNA) identified several gene modules and their hub genes, including ion transport related modules consisting of genes that negatively regulate N uptake including *OsHHO3*, *OsBT*, and *OsACTPK1* in all the lines. The repression of these genes under low N could be a basic mechanism to facilitate N acquisition in rice roots. The network analysis also identified cell activity and cell wall modification modules in the introgression lines which could be coordinated by *OsLBD3-1*, a paralogue of the *Crown rootless*1 gene for the promotion of root development to enhance N acquisition under low N conditions. The present analysis revealed the involvement of major pathways for low nitrogen tolerance of the selected lines.

## 1 Introduction

Nitrogen (N) is one of the most important nutrients for plant growth and development. The yield of crops highly depends on the plant nitrogen status, which is mostly fulfilled through the application of nitrogenous fertilizers in the field (Ågren and Weih, 2012). The excessive use of these fertilizers not only adds to the cost of crop production but also creates environmental pollution; therefore, the breeding of crop varieties with high nitrogen use efficiency is essential. Rice (*Oryza sativa* L.) is an important food crop, and achieving a high nitrogen use efficiency (NUE) in rice is a target of much research since loss of fertilizer from rice fields significantly contributes to pollution, including eutrophication (Coskun et al., 2017; Yu et al., 2019). Although single-gene transgenic interventions have been able to make some improvements to NUE component traits in various model crops, the plant NUE still remains a highly complex trait that requires coordination of several processes both at molecular and physiological levels (Xu et al., 2012). It requires the development of varieties with improved root systems for efficient nutrient uptake, root-to-shoot transport, and mineral remobilization under low nutrient conditions.

Several genes control N metabolism pathways from uptake to utilization. During N assimilation, NO_3_^−^ and NH_4_^+^ taken up by plants through transporters are used to synthesize it to glutamine (Gln) using glutamine synthetase (GS) through the GS/glutamate synthase (GOGAT) cycle (Yamaya and Oaks, 2004; Neeraja et al., 2021). Therefore, different N regimes and varietal difference influence the expression of these processes and the genes involved in the N metabolism pathway, resulting in differential N responses. In rice, the modulation of transporter genes was reported to have increased plant biomass and seed yield (Bi et al., 2009; Peng et al., 2014). The overexpression of *OsAMT1.1* increased NH_4_^+^ uptake, plant growth, grain filling, and total number of grains per plant, specifically under low NH_4_^+^ conditions in rice (Ranathunge et al., 2014). The genetic alteration of transcription factors (TFs) that simultaneously regulate a number of N use related genes also appears to be one of the promising strategies to improve N use efficiency according to Ueda and Yanagisawa (2018). Moreover, the genotypic difference in the regulation of nutrient uptake and other physiological processes in response to nutrient availability have been observed among genetically diverse cultivars of several crop plants, including rice, resulting in varying NUE among cultivars (Famoso et al., 2011; Hu et al., 2015; de Abreu Neto et al., 2017). This is because plants modulate their physiological traits such as root system architecture and transcriptome makeup, including N uptake and assimilation genes, to adapt to the amount of N available in the soil (Giehl et al., 2014). Therefore, transcriptome analysis of rice cultivars with diverse genetic backgrounds may shed light on differential regulatory response among various lines.

Genes that co-expressed tend to participate in similar biological processes; hence, their expression could be controlled by similar regulatory mechanisms (Michalopoulos et al., 2012; Petereit et al., 2016). This comprises of transcriptional regulatory networks that allow plants to integrate various types of signals generated by sensing internal and external factors, transmitting the integrated information to regulatory systems for diverse biological processes and coordinately regulating these processes (Rouached and Rhee, 2017). Weighted gene co-expression network analysis (WGCNA) uses system biology to understand networks of genes that co-express and explore the associations between genes and the target traits (Langfelder and Horvath, 2008). In rice, WGCNA was employed to identify core genes regulating the response to abiotic stress such as drought, cold, salinity, and nitrogen metabolism (Lv et al., 2019; Zhu et al., 2019; Ueda et al., 2020). Gene co-expression analysis revealed gene networks related to secondary metabolite biosynthesis and carbohydrate metabolism and provided insights into the relationship between C/N balance and cell wall biogenesis in several crops (Zhao et al., 2015; Goel et al., 2018). It was reported that plants utilize a large proportion of their photosynthetic products to generate cell wall polymers for growth, directly associating carbon metabolism with cell wall biosynthesis, which must coordinate with nitrogen metabolism (Gao et al., 2020). In rice and other plants, N deficiency stress was reported to affect the expression of some cell wall-related genes, such as expansins, suggesting a link between cell wall biogenesis and C/N balance response (Scheible et al., 2004; Rivai et al., 2021).

In the current study, RNA-Seq analysis has been applied on hydroponically grown rice seedlings of Koshihikari (KH) and its nitrogen use efficient introgression lines (KRIL8 and KRIL37) to dissect the transcriptome changes in the shoot and roots under low (0.4 mM NH_4_^+^) and normal (1.6 mM NH_4_^+^) N conditions at several time points. These introgression lines that carry a small region of the wild rice *Oryza rufipogon* genome in the KH background were reported to be nitrogen use efficient under limited nutrient conditions (Adu et al., 2022) and showed bigger above- and below-ground biomass under low N conditions. The transcriptome analyses revealed differential expressions of N and C metabolism related genes in response to low N conditions among the lines. WGCNA was performed and identified critical groups of co-expressed genes involved in ion transmembrane transport, cellulose biosynthesis, cell wall remodeling, and their hub genes in response to N deficiency, which may act as key regulators in variable response to low N conditions among the genotypes. The study highlights the potential of low N stress induced ion transport which could be coordinated by an *OsHHO3* (a member of the NIGT1/HHO subfamily of the GARP/G2-like transcription factor family) and cell wall biogenesis processes potentially regulated by a *Crown Rootless-Like* gene (*OsCRLL1*, *OsLBD3-1*) to drive root elongation in the nutrient-efficient lines for nutrient exploration.

## 2 Materials and methods

### 2.1 Plant materials

The wild rice introgression lines (ILs, cultivated rice KH with introgressed genomic regions from the wild species *Oryza rufipogon*) were kindly provided by Dr. Hideyuki Hirabayashi (Hirabayashi et al., 2010). Following field initial evaluation of these ILs under non-fertilized paddy field conditions in Fukushima (Japan), KRIL8 and KRIL37 were selected based on panicle yield (Adu et al., 2022). The recurrent parent Koshihikari (KH) was part of our laboratory stock.

### 2.2 Hydroponics cultivation

Seeds were incubated at 45°C for 4 days to break dormancy before using them for germination. The seeds were surface sterilized with 10 times diluted bleach (6% hypochlorite solution) for 30 min and washed with distilled water. Then, seeds were incubated on filter papers with distilled water until germination at 29–30°C inside a controlled growth chamber (for approximately 5 d). Germinated seeds were transferred to dark-colored 3-L polyethylene containers with liquid media of different conditions. Modified Kimura B solution (Tanaka et al., 2018) was used as the base of the N-free culture media [NH_4_Cl, K_2_SO4, and CaSO_4_·2H_2_O were used in place of (NH_4_)_2_SO_4_, KNO_3,_ and Ca(NO_3_)_2_·4H_2_O, respectively]. To create different N growth conditions, 0.4 mM and 1.6 mM NH_4_Cl were used in the experiment as low N (LN) and normal N (NN), respectively. Hydroponic cultivation was done in a growth chamber (NK System Biotron, Nippon Medical and Chemical Instrument, Japan) at 29–30°C with ~69% RH under fluorescent lamps (14.5 h light and 9.5 h dark cycles) for two weeks. After one week of growth in the NN condition, uniform size seedlings were transferred to fresh LN (0.4 mM NH_4_^+^) and NN (1.6 mM NH_4_^+^) treatment media. The shoot and root tissues were harvested after 12 h, 24 h, 48 h, 96 h, and 168 h of treatments and measured for shoot and root length, shoot and root dry weight, and shoot and root N%.

For dry weight and tissue N% determination, samples were dried in an oven at 70°C for at least 4 days. To determine the tissue N content, dried samples were weighed (≤100 mg) in crucible containers for combustion using a CN elemental analyzer (vario MAX cube, Elementar, Kanagawa, Japan).

Estimation of N use efficiency parameters was done at 168 h where N deficiency was highest. NUpE (N uptake efficiency) was estimated as tissue N/N in nutrient solution, while NUE (N use efficiency) was estimated as dry weight/N in the nutrient solution (Moll et al., 1982).

### 2.3 RNA extraction and transcriptome analysis

The root and shoot tissues from each genotype were collected separately at each time point (12 h, 24 h, 48 h, 96 h and 168 h) and immediately frozen in liquid nitrogen, and stored at −80°C. Total RNA was extracted from the tissues using RNeasy Plant Mini Kit (Qiagen, Tokyo, Japan). For all time points, both treatments and all genotypes, RNA was extracted from three biological replicates from both tissues (180 libraries). Total RNA was quantified using a Qubit RNA BR Assay Kit and Qubit 4 Fluorometer (Thermo Scientific, USA). Library preparation and transcriptome sequencing were conducted by Clockmics (Clockmics, Inc., Osaka, Japan) using Illumina platform Hiseq X to generate 150 long paired-end reads.

Read processing and transcript quantification were done as follows: Trimmomatic (Bolger et al., 2014) was used to trim (discard) low-quality reads, trim adaptor sequences, and eliminate poor-quality bases to maximize the value of each read and prevent aligning reads to wrong positions against the reference. Cleaned reads were aligned to the rice reference genome assembly (Oryza_sativa.IRGSP1.0.dna_sm.toplevel.fa.gz) from EnsemblPlants (https://ftp.ensemblgenomes.ebi.ac.uk/pub/plants/release-57/fasta/oryza_sativa/dna/) using Hisat2 (v2.2.1) (Kim et al., 2015) with default parameters. To obtain expression data, mapped reads were counted for each gene by featureCounts (Liao et al., 2014) with default parameters using Oryza_sativa.IRGSP-1.0.52.chr.gtf from EnsemblPlants as a gene annotation file.

Raw read counts were normalized using trimmed M-means in edgeR (Robinson et al., 2009). Differential expression genes (DEGs) analysis across samples was conducted using the edgeR package to obtain differently expressed genes (DEGs) at FDR (false detection ratio) p ≤ 0.05, and log2 FC ≥ |1| for upregulated and downregulated genes (Benjamini and Hochberg, 1995). Biologically meaningful comparisons were made between plants grown under LN and NN conditions within each genotype at each time point. The terms upregulated and downregulated were used to refer to the expression values of LN samples relative to the NN expression values.

### 2.4 Co-expression network analysis

Weighted gene co-expression network analysis (WGCNA) was conducted using R with the WGCNA package (Langfelder and Horvath, 2008). The DEGs obtained at each time point in both root and shoot tissues were used for the network analysis in each genotype. In network analysis, a correlation between all gene pairs was calculated to establish a similarity matrix. The pickSoftThreshold function was used with the following options: networkType = “unsigned”, RsquaredCut = 0.8. Parameters to detect the module were as follows: minClusterSize = 30 in cutreeDynamic function, cutHeight = 0.20 in mergeCloseModules function. The default parameters were used for others. A corresponding module color was assigned to each gene if its co-expression partners could be defined using the abovementioned criteria. To identify modules that are significantly associated with tissue N, a correlation analysis of module eigengenes and phenotypic data was done. Modules with significantly positive correlation (r ≥ 0.5, p ≤ 0.05) with shoot or root N were extracted for further analysis.

Upon identifying the modules, biological interactions within the module genes were analyzed using STRING v12.0 (https://string-db.org/). The protein–protein interaction (PPI) enrichment in each module was calculated using built-in algorithms of the STRING and displayed by Cytoscape software. The highly connected genes identified from PPI networks (node degree) were considered as hub genes essential for physiological functions (Zhang et al., 2018). Using Cytoscape (v3.9.1), the interactions within the modules for the top 30–100 genes were visualized using the output files from STRING (Shannon et al., 2003). In the network, the DEGs with the top 10 weight values (node degree) including transcription factors with >10 node degrees were identified as candidate genes denoted by turquoise-blue color in the network.

### 2.5 Validation of RNA-Seq data by qRT-PCR

About 300 ng of total RNA was reverse-transcribed in a 10-μL reaction using PrimeScript RT reagent kit (Takara Bio, Kusatsu, Japan) with oligo-dT_16_ primers. The cDNA was amplified by qRT-PCR using Thermal Cycler Dice Real Time System TP800 (Takara Bio, Kusatsu, Japan) with SYBR Premix Ex Taq kit (Takara Bio, Kusatsu, Japan). qPCR was performed in triplicate for each sample, and the relative expression levels were normalized to that of *Actin1* (*Os03g0718100*) gene. The primers used for qRT-PCR are listed in **Supplementary Table 1**.

### 2.6 Data visualization and enrichment analysis

The heatmap was generated using the “*pheatmap*” package in R (Kolde, 2022) while the interaction between the module genes were visualized using Cytoscape (v3.9.1) (Shannon et al., 2003). To analyze the functional annotation of the identified DEGs and module genes, all genes in the candidate modules were subjected to gene ontology (GO) functional annotation and Kyoto Encyclopedia of Genes and Genomes (KEGG) pathway enrichment analyses using the ShinyGO v0.77 (Ge et al., 2020) with FDR ≤ 0.05 as the threshold to identify the significant biological pathways.

## 3 Results

### 3.1 Early growth response of the rice lines sample across time

**Figure 1:**
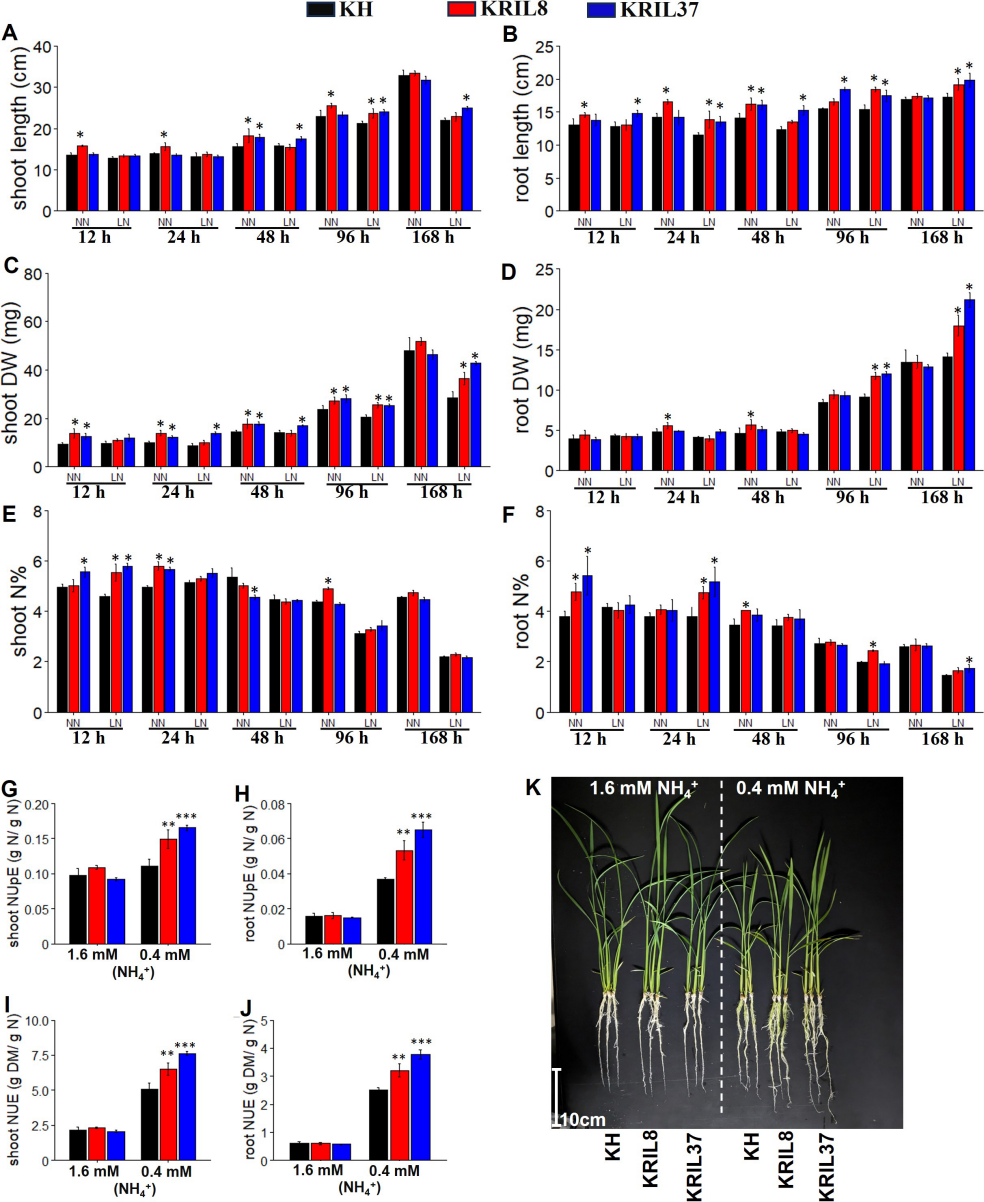
Seedling growth performance of the rice KH, KRIL8, and KRIL37 under hydroponic evaluation. The seedling characteristics of KH, KRIL8, and KRIL37 grown in modified Kimura B solution at 0.4 mM NH_4_^+^ (LN) and 1.6 mM NH_4_^+^ (NN) sampled across different time points **(A–F)**. Shoot length **(A)**, root length **(B)**, shoot dry weight **(C)**, root dry weight **(D)**, shoot N percent **(E)**, and root N percent **(F)**. Nitrogen use efficiency parameters of KH, KRIL8, and KRIL37 computed at 168 hours of sampling **(G–J)**. **(G)** Shoot NUpE, **(H)** root NUpE, **(I)** shoot NUE, and **(J)** root NUE. NUpE, nitrogen uptake efficiency (shoot N/N in solution); NUE, nitrogen use efficiency (dry shoot biomass/N in solution). Bars with *, **, and *** are significant at p<0.05, 0.01, and 0.001 respectively with KRIL8 or KRIL37 compared to KH using Dunnett’s test. Error bar = SD, n = 4–5.

To understand the phenotypic response of the lines to two ammonium concentrations (0.4 mM NH_4_^+^ as low N, LN; and 1.6 mM NH_4_^+^ as normal N, NN), growth parameters such as shoot and root length, shoot and root dry weight, and shoot and root N concentration were measured at each time point (**Figure 1**). The ILs (KRIL8 and KRIL37) continuously gave higher root and shoot traits across the sampled time points, indicating their relative ability to produce carbon under deficient N conditions. Shoot and root length increased under LN in the ILs after 7 days of exposure (**Figures 1A, B**). The KRIL8 and KRIL37 recorded over 30% increase in shoot and root dry weight over KH after 168 hours of LN treatment (**Figures 1C, D, K**). The enhanced root biomass could influence the efficient N accumulation into tissue (uptake) for better growth under low N stress conditions. Also, the accumulation of N into the shoot and root tissues tended to be higher in the ILs. The tissue N content was significantly higher in the ILs during the first 24 hours of treatment in the shoot and toward the later stage (96–168 h) in the root (**Figures 1E, F**).

The tissue dry weight was positively associated with the nitrogen use efficiency (NUE) assessed among the lines. Nitrogen uptake efficiency (NUpE), representing the uptake of nitrogen from the nutrient solution, was higher in the ILs compared to KH at the last sampling point (168 h, where N limitation was highest), suggesting that nitrogen uptake was enhanced in the ILs, and this resulted in increased overall NUE (dry biomass/available N) in the ILs compared to KH (**Figures 1G–J**).

### 3.2 Differential gene expression analysis and GO analysis across the sampled time

To understand the global transcriptional response of the rice lines in response to LN and NN ammonium levels treatments, RNA-Seq analysis was performed for the whole shoot and whole root tissues sampled across five different time points (12 h, 24 h, 48 h, 96 h, and 168 h) for each line. The PCA analysis revealed a strong grouping of libraries based on the sampled time point indicating that the length of exposure to the treatments significantly influenced the expression of the genes and high reproducibility (**Supplementary Figure 1**).

The differential gene expression analysis was conducted between LN and NN conditions at each time point (12 h, 24 h, 48 h, 96 h, and 168 h) separately in the shoot and root tissues within each genotype (DEGs were selected at log2 FC ≥ |1|, FDR: p ≤ 0.05) using edgeR (Robinson et al., 2009). The number of DEGs obtained at each time point (**Table 1**) shows an increasing number of DEGs as the duration of N stress treatment progresses, suggesting a gradual response toward low N stress. To validate the RNAseq data, four DEGs were randomly selected for qRT-PCR assay (**Supplementary Figure 2**). As anticipated, a similar expression pattern was observed between the qRT-PCR results and RNAseq data as confirmed by a Mantel test (r = 0.90, p ≤ 0.001), indicating the credibility of the RNA-Seq data. Enrichment analysis was carried out to identify the biological processes and pathways that were significantly enriched by the identified upregulated or downregulated genes in each genotype across the sampled time (**Supplementary Tables 2**-**4**).

**Table 1:** Number of differently expressed genes (DEGs) (FDR > 0.5, (log2 FC |≥1|) in the shoot and root tissues of the cultivars at each time point (UP = upregulated, DOWN = downregulated).

SHOOT	12 hours		24 hours		48 hours		96 hours		168 hours		Total
	UP	DOWN	UP	DOWN	UP	DOWN	UP	DOWN	UP	DOWN	
KH	204	253	1	3	610	855	563	1050	304	2071	5445
KRIL8	24	19	0	6	62	260	233	664	1254	1868	3749
KRIL37	761	708	57	34	377	514	141	279	1396	2321	5493

ROOT	UP	DOWN	UP	DOWN	UP	DOWN	UP	DOWN	UP	DOWN	Total
KH	147	367	63	99	605	167	866	694	1938	2443	5784
KRIL8	17	110	284	424	197	104	532	596	2747	2550	6335
KRIL37	2	21	32	46	102	34	562	522	989	1396	3000

Analysis revealed that biosynthesis of secondary metabolites (GO:0044550) was enriched in the shoot of all the lines within the first 12 hours of introduction to low N condition as well as toward the later stage (96–168 h), which included phenylpropanoid biosynthesis (KEGG:00940), diterpenoid biosynthesis (KEGG:00904), and response to oxidative stress (GO:0006979) in the root. At the height of N deficiency (96–168 h), cell nuclear activities in the shoot involving ribosome such as translation (GO:0006412) and peptide biosynthesis (GO:0043043) stalled in all the lines including the structural constituent of the ribosome (GO:0042254), which is the hub of amino acids production (**Supplementary Tables 2**-**4**). This resulted in the downregulation of photosynthesis (KEGG:00195) and reduced growth under low N stress.

However, the GO analysis also revealed the differential response of the lines to low N stress across the sampled time. Interestingly, processes such as chromatin remodeling (GO:0006338) including several histones, DNA replication, and cell cycle (GO:0022402) were significantly downregulated in the shoot of KH within the first 12 hours of introduction to low N conditions (**Supplementary Table 2**). In plants, the epigenetic regulation of abiotic stress includes exploitation of chromatin modification mechanisms to adapt to its surrounding environment (Asensi-Fabado et al., 2017). This suggests that the nutrient stress condition significantly impacted KH earlier while the tolerant lines KRIL8 and KRIL37 appear to be transcriptionally stable. During abiotic stress, ROS accumulates as a result of oxidative stress, which is dealt with by an antioxidant defense system in plants (Khan et al., 2023). The nitric oxide (NO) biosynthetic process (GO:0006809) was induced in the shoot of KRIL37 at the peak of N deficiency (168 h), which could be a low N stress signaling response strategy as well as defense mechanism against cell damage by oxidative stress.

### 3.3 Differential expression of N and C metabolism genes

**Figure 2:**
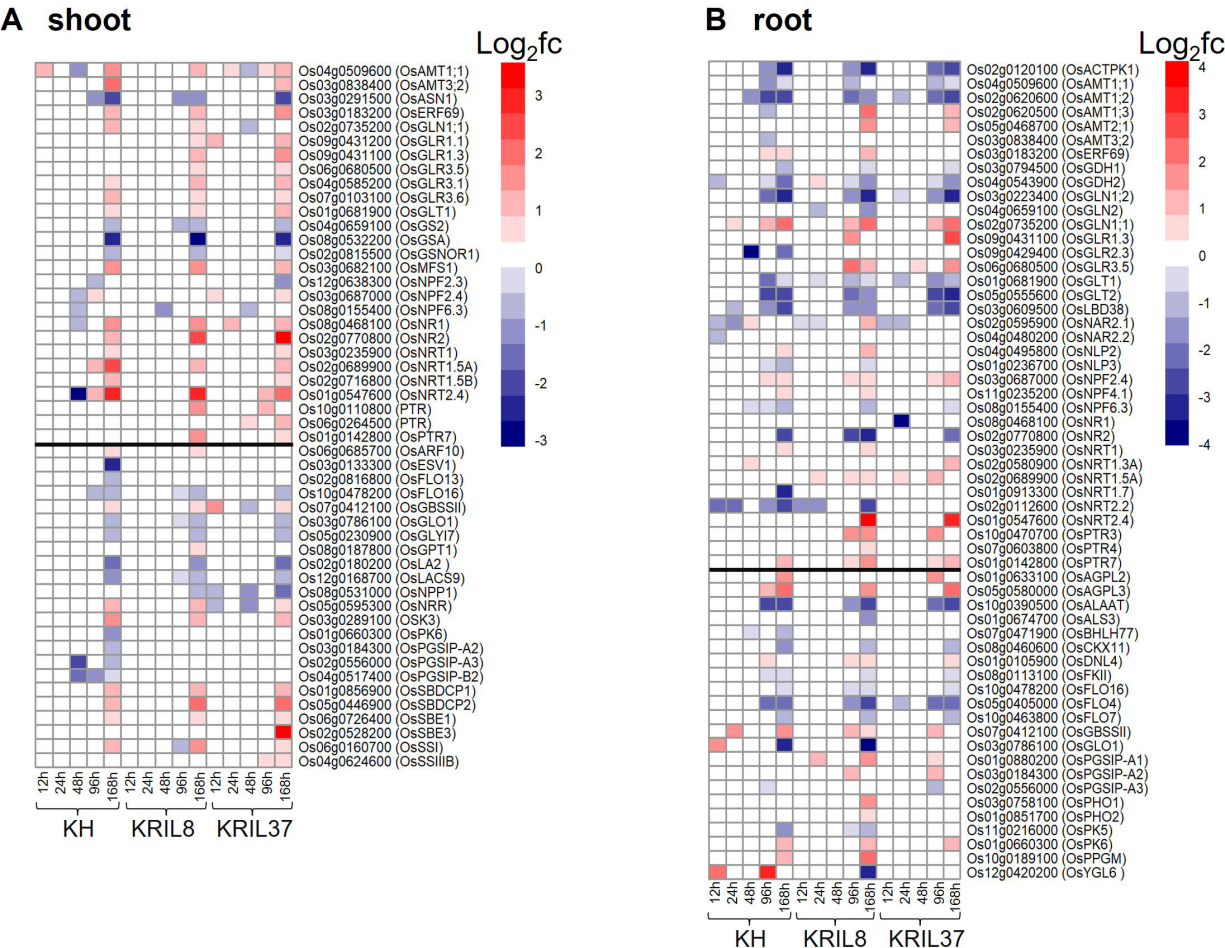
Heatmap shows relative expression of N utilization and carbon metabolism related genes under low nitrogen (LN) conditions compared to normal nitrogen (NN) in each line. Heatmap showing differently expressed genes (DEGs) identified under LN conditions in comparison to NN in each genotype that are associated with N uptake, N assimilation, and carbohydrate metabolism in the **(A)** shoot and **(B)** root. The black line separates N utilization related genes from carbohydrate metabolism related genes. The vertical bar indicates relative expression ratio in log2 FC [DEGs were selected at FDR (p ≤ 0.05), and log2 FC ≥ |1| for upregulated and downregulated genes]. Gene code and gene symbol for each gene can be found at the Rice Annotation Project Database (https://rapdb.dna.affrc.go.jp/).

Among the enriched GO and pathway terms, photosynthetic processes (KEGG:00710) and nitrogen metabolism (KEGG:00910) were identified to be associated with N and carbon (C) metabolism. Differently expressed genes (DEGs) identified under low nitrogen (LN) conditions in comparison to normal nitrogen (NN) in each genotype which are reported to be involved in N and C metabolism (according to https://rapdb.dna.affrc.go.jp) were compared among the lines in both shoot and root tissues in a heatmap (**Figure 2**). Overall, most of the genes showed differences in expression between LN and NN conditions toward the later part of N stress (96–168 h).

The expressions of some of the genes were similar among the three genotypes. The pattern of expression of N uptake genes such as *OsAMT1;1*, *OsNRT1.5A*, and *OsNRT2.4* were similar in the shoot among the genotypes (**Figure 2A**). These genes were upregulated during the peak of N stress (168 hours). In the root, *OsACTPK1*, *OsAMT1;1*, *OsAMT1;2*, *OsGDH1*, *OsGDH2*, *OsGLN1;2*, *OsGLT1*, and *OsGLT2* showed a similar downregulation response among the genotypes under LN (**Figure 2B**). Genes involved in the regulation of initial carbon production (chlorophyll biosynthesis and photosynthesis) such as *OsFLO16*, *OsGLO1*, *OsGLYI7*, and *OsLA2* were downregulated in a similar trend among the lines in the shoot. On the contrary, genes such as *OsGBSSII*, *OsSSI*, *OsNRR*, and *OSK3* and starch binding domain-containing proteins (*OsSBDCP1* and *OsSBDCP2*) involved in starch biosynthesis and accumulation were significantly expressed under low N stress (**Figure 2A**). These genes could be involved in direct biomass accumulation and photosynthetic activity.

In spite of the above findings, significant variation was also observed in the expression of N and C metabolism related genes in the tissues of the different lines. Ammonium and nitrate transporters *OsAMT1;3*, *OsAMT2;1*, *OsNRT1.5A*, and *OsNRT2.4* were significantly upregulated in the roots of ILs (KRIL8 and KRIL37) not in KH toward the end of low N stress. In the shoot, peptide transporter family members *OsPTR3* and *OsPTR7* were significantly upregulated in the shoot of the ILs (**Figure 2A**). These transporters are involved in N uptake and accumulation in the tissues, which could influence the differential adaptation to low N stress among the genotypes. The role of glutamate receptors in nutrient metabolism (Ca^2+^, N) and root morphology is well documented (Toyota et al., 2018). In nitrogen-starved rice seedlings, glutamate was the most abundant free amino acid and could be easily metabolized and converted to the other nitrogen-containing compounds in rice (Kan et al., 2017; Li et al., 2019). Here, glutamate receptors (*OsGLR1.1*, *OsGLR1.3*, and *OsGLR3.5*) were highly expressed under low N in the shoot and roots of the ILs compared to KH. A group of starch and cellulose metabolism genes annotated as plant glycogenin-like starch initiation protein (PGSIP) including *OsPGSIP-A2*, *OsPGSIP-A3*, and *OsPGSIP-B2* showed significantly reduced expression in the shoot of KH toward the end of N stress, but this was not the case in the ILs (**Figure 2A**). In the root, *OsPGSIP-A1* and *OsPGSIP-A2* responded positively to low N in the ILs (**Figure 2B**). The differential activation of genes involved in N transport, assimilation, and carbon metabolism could potentially influence genotypes adaptation to low N stress.

### 3.4 Construction of gene co-expression network (modules)

**Figure 3:**
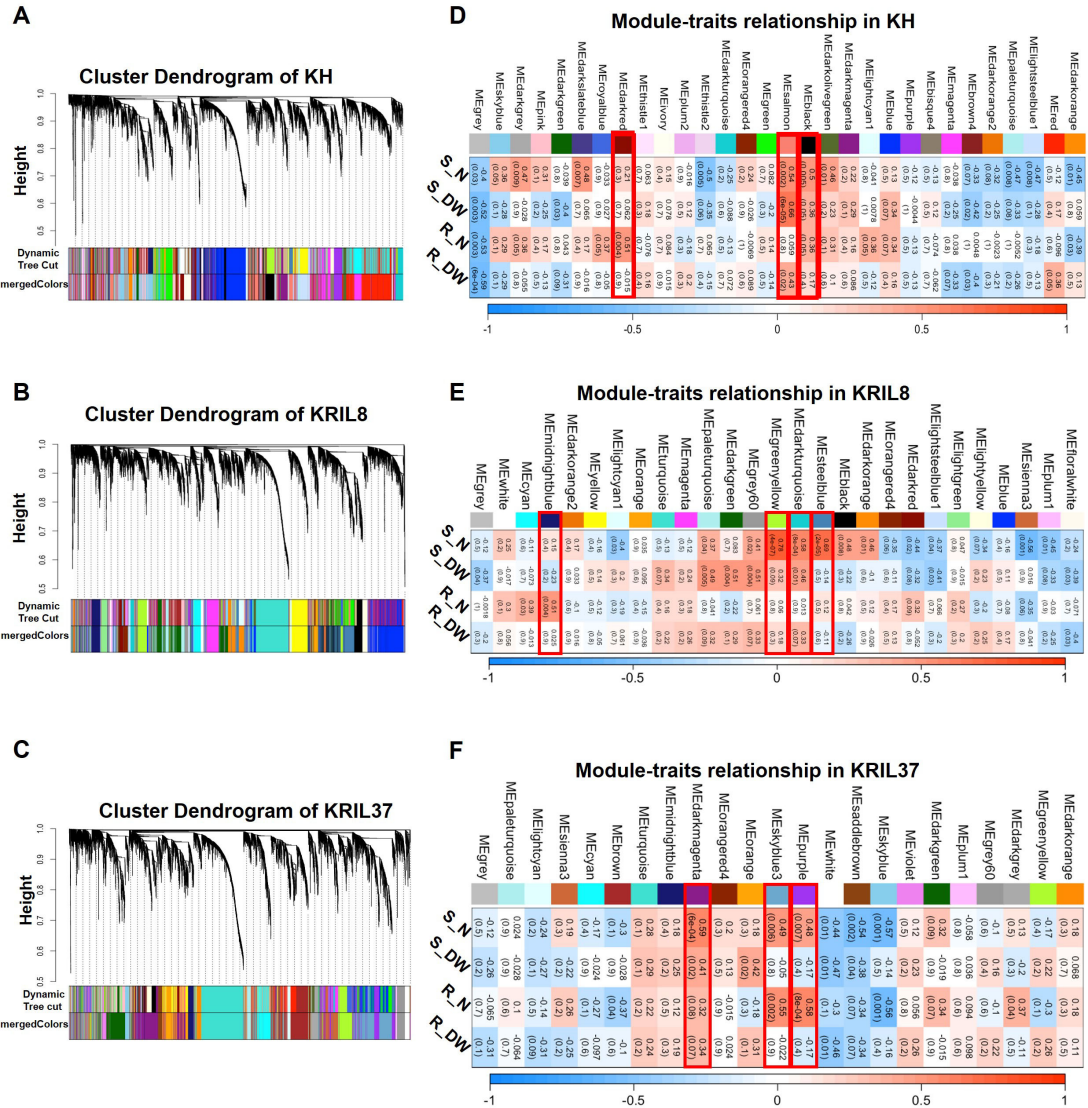
Weighted gene co-expression network analysis of DEGs into modules (colors). Hierarchical cluster tree showing dendrogram of DEGs and the assignment of co-expressed genes into modules (represented by colors) identified by weighted gene co-expression network analysis (WGCNA). Each leaf in the cluster tree represents one gene. The color strips were used for simple visualization of module assignment of each gene in **(A)** KH, **(B)** KRIL8, and **(C)** KRIL37. Module–traits relationship and significant modules identified in KH, KRIL8, and KRIL37 by WGCNA **(D–F)**. **(D)** KH, **(E)** KRIL8, and **(F)** KRIL37. The modules highlighted with red border box represent significantly enriched modules associated with shoot nitrogen (S_N) or root nitrogen (R_N) concentration in each genotype. For values in the table, the top value is the corresponding correlation coefficient between the module eigengene and the phenotypic traits while the bottom in parentheses is p-value.

Genes that co-express tend to participate in similar biological processes; hence, their expression could be controlled by similar regulatory mechanisms (Petereit et al., 2016). Weighted gene co-expression network analysis (WGCNA) was used to further understand low N stress regulatory network by identifying genes that co-express as modules in each line (Langfelder and Horvath, 2008). The 11,229, 10,084, and 8493 individual DEGs obtained in KH, KRIL8, and KRIL37, respectively (**Table 1**), were used to build the gene networks in each line. The genes demonstrated hierarchical clustering according to the TOM-based dissimilarity (1-TOM) measure (Langfelder and Horvath, 2012). After hierarchical clustering, highly interconnected genes were assigned to the same module (denoted by colors) while gray consisted of genes without assignment to any module (**Figure 3**). Modules with correlation of module eigengenes higher than 0.8 were merged, resulting in 29, 27, and 23 modules for KH, KRIL8, and KRIL37, respectively (**Figure 3**).

Previous physiological and biochemical studies revealed that N deficiency decreases the photosynthetic efficiency of the plant although this condition could be recovered upon N supplementation, indicating that cellular C and N metabolism is tightly coordinated in plants (Coruzzi and Zhou, 2001). To identify gene modules that are significantly associated with the phenotypic traits such as shoot and root N% and dry weight (DW), a correlation analysis of module eigengenes and these phenotypic traits was done. Using positive correlation co-efficient (r ≥ 0.5) between a gene module and tissue N%, three modules in KH (Black, Darkred and Salmon), four in KRIL8 (Darkturquoise, Greenyellow, Midnightblue, and Steelblue), and three in KRIL37 (Darkmagenta, Purple, and Skyblue3) were found to be significantly (p ≤ 0.05) associated with the tissue N and were selected for further analysis (**Figure 3**).

### 3.5 Enrichment analysis of the module genes and identification of highly connected genes

**Figure 4:**
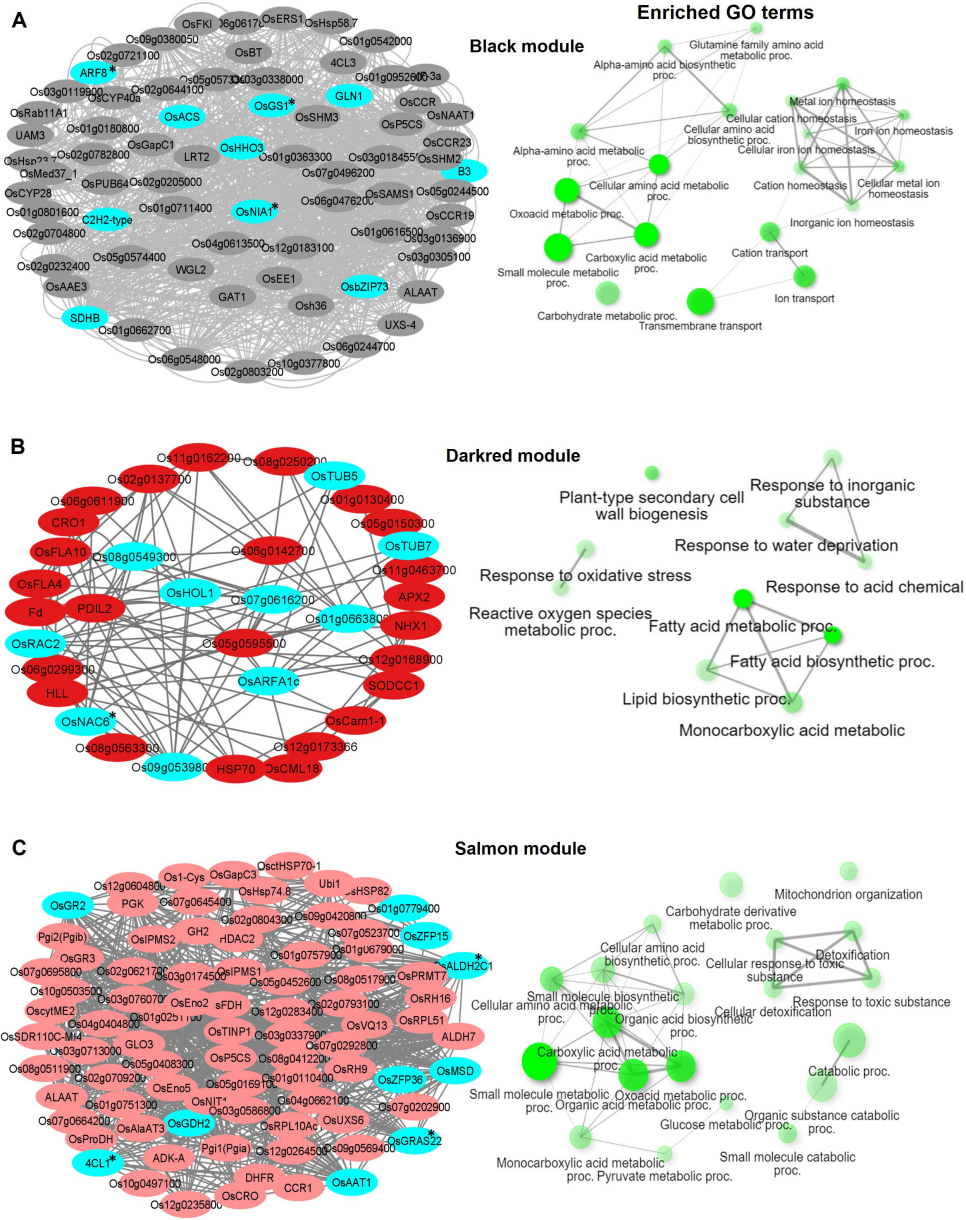
Visualization of connections of top genes in various modules of KH. Gene network analysis and GO terms of **(A)** Black module, **(B)** Darkred module, and **(C)** Salmon module. Turquoise-blue colored nodes suggest their central role in the network. Nodes with asterisks indicate upregulated central key genes. The network of biological processes (GO) that the genes are involved in is indicated on the right of the gene network.

**Figure 5:**
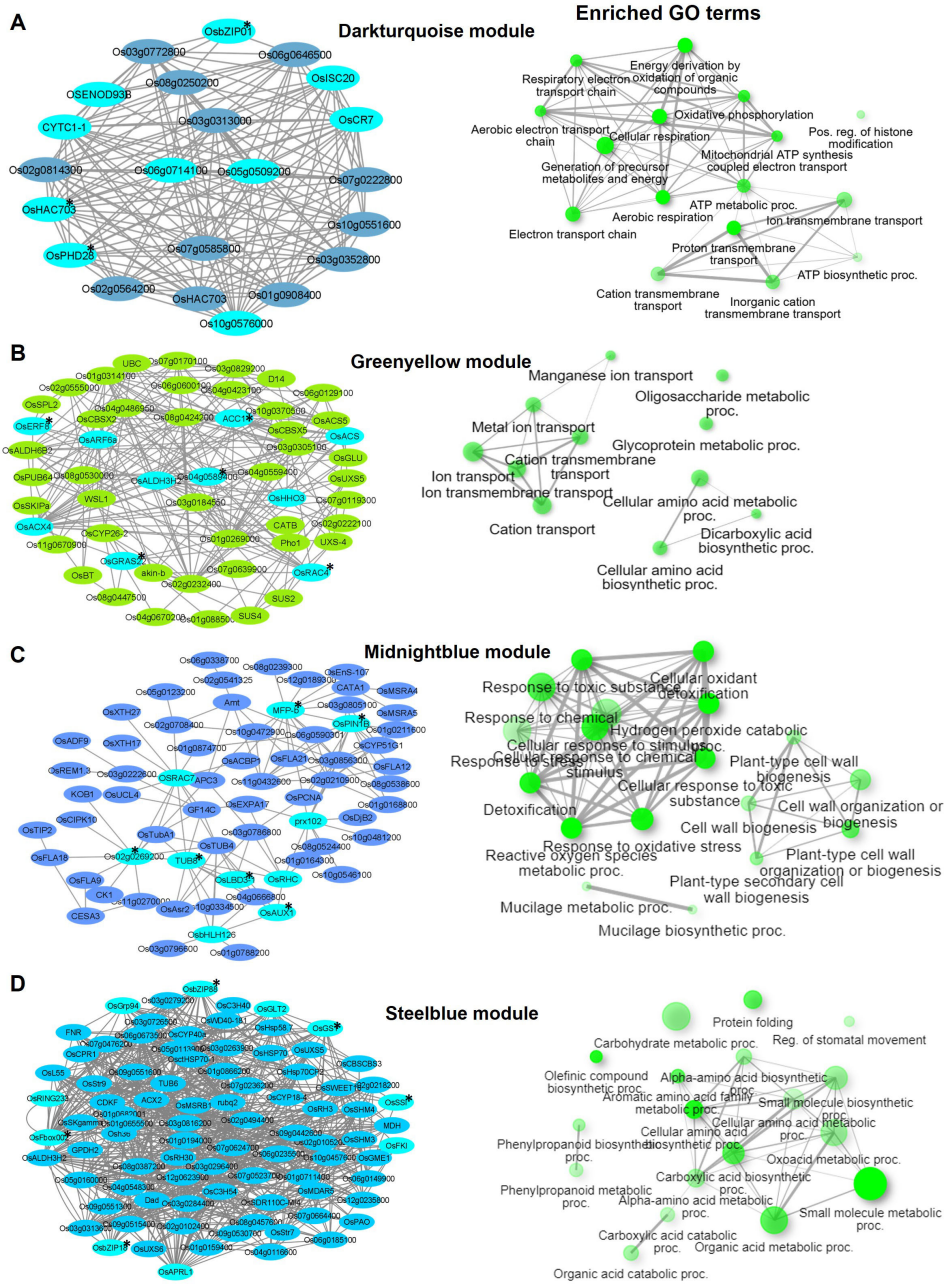
Visualization of connections of top genes in various modules of KRIL8. Gene network analysis and GO terms of **(A)** Darkturquoise module, **(B)** Greenyellow module, **(C)** Midnightblue module, and **(D)** Steelblue module. Turquoise-blue colored nodes suggest their central role in the network. Nodes with asterisks indicate upregulated central key genes. The network of biological processes (GO) that the genes are involved in is indicated on the right of the gene network.

**Figure 6:**
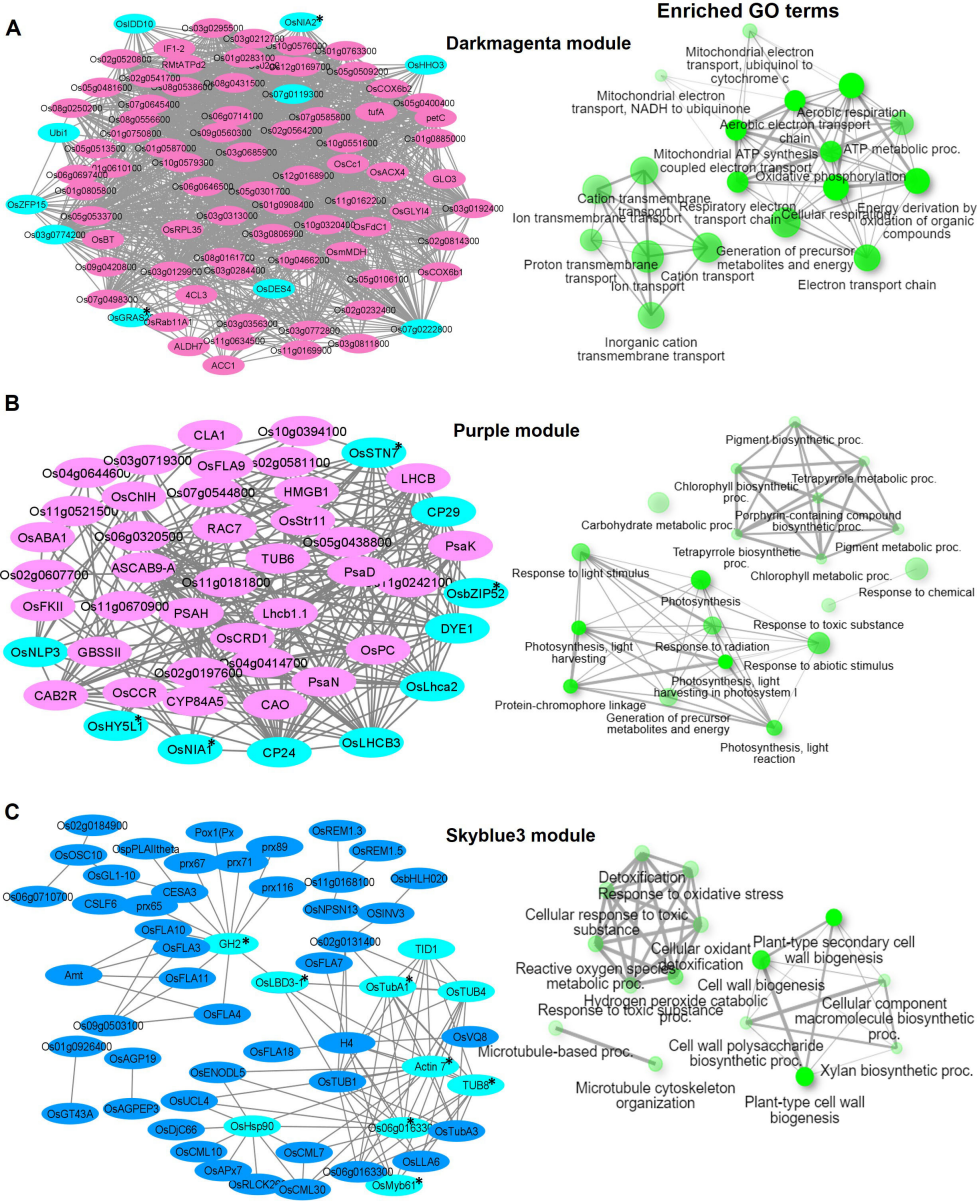
Visualization of connections of top genes in various modules of KRIL37. Gene network analysis and GO terms of **(A)** Darkmagenta module, **(B)** Purple module, and **(C)** Skyblue3 module. Turquoise-blue colored nodes suggest their central role in the network. Nodes with asterisks indicate upregulated central key genes. The network of biological processes (GO) that the genes are involved in is indicated on the right of the gene network.

Gene ontology enrichment analysis of each of the modules in each genotype revealed several significant terms (FDR < 0.05) (**Figures 4**-**6**). To identify the hub genes in each module, the top highly connected genes (high node degree) of each module were obtained after ordering the node degree in the network (module). The top highly connected genes and transcription factors (≥10 node degree) were considered as central genes in each module. The top 30–100 connected genes of each module were analyzed and visualized through Cytoscape (Shannon et al., 2003) to draw the network diagram (**Figures 4**-**6**).

In KH, the Black module (746 genes) was highly enriched in transmembrane transport (GO:0055085), carboxylic acid metabolic process (GO:0019752), and ion transport (GO:0006811) biological terms (**Figure 4A**). Under nitrogen stress, maintaining the correct balance of other ions (Ca, K, Na) inside and outside the cell is essential for nitrogen metabolism. Several ion transporters such as *OsAMT1* (ammonium transporter) and *OsHKT1* (Na+/K+ symporter) were enriched in this module. *OsNIA1*, *OsACS*, *OsHHO3* (a member of the NIGT1/HHO subfamily of the GARP/G2-like transcription factor family), and *OsDLN129* (zinc finger, C2H2-type domain containing protein) were among the top genes that highly co-expressed with other genes in this module (**Figure 4A**). In rice, *OsNIA1* and *OsHHO3* are reported to significantly influence NUE (Gao et al., 2019; Liu et al., 2023). The Darkred module (300 genes) of KH was associated with lipid biosynthetic process (GO:0008610), fatty acid metabolic process (GO:0006631), and monocarboxylic acid metabolic process (GO:0032787) and consisted of several fatty acid 2-hydroxylases including *OsFAH1*, *OsFAH2*, and *OsSPH* (**Figure 4B**). Nitrogen starvation is reported to increase lipid accumulation in organisms like microalgae and fungi (Li et al., 2024). The third module Salmon (677 genes) had small molecule metabolic process (GO:0044281), organic acid metabolic process (GO:0006082), cellular amino acid metabolic process (GO:0006520), and pyruvate metabolic process (GO:0006090) as the top GO terms (**Figure 4C**).

In KRIL8, generation of precursor metabolites and energy (GO:0006091), ion transmembrane transport (GO:0034220), and cellular respiration (GO:0045333) were the top GO terms in the Darkturquoise module (**Figure 5A**). The Greenyellow module (477 genes) was enriched in cellular amino acid metabolic processes (GO:0008652), ion transport (GO:0006811), cation transport (GO:0006812), and ion transmembrane transport (GO:0034220) (**Figure 5B**). The Midnightblue module (251 genes) was associated with response to oxidative stress (GO:0006979), cell wall biogenesis (GO:0071554), and plant type and cell wall organization (GO:0071669) terms (**Figure 5C**). Recent studies revealed that N deficiency results in changes in cell wall composition, including alterations in polysaccharide content and cell wall thickness *Ganoderma lucidum*, directly implicating N metabolism in cell wall organization (Shi et al., 2025). *OsbHLH126* (bHLH transcription factor) and *OsLBD3-1* (lateral organ domain protein, *CRL1-LIKE 1*) involved in the regulation of root hair development were the main TFs in this module. Taken together, the Midnight module could be involved in cell expansion and organ growth under N stress. In addition to the cell wall related terms, the Midnightblue module of KRIL8 specifically consisted of a cellular response to stimulus (GO:0051716) and signal transduction (GO:0007165) GO terms made up of several auxin transport and signaling genes (*OsPIN1B*, *OsAUX1*, *OsIAA6*, and *OsSAUR21*) and serine/threonine kinases including those involved in brassinosteroid signaling (*OsBRI1*), which could modulate N signaling transduction in KRIL8 (**Figure 5C**). Protein folding (GO:0006457), carbohydrate metabolic process (GO:0005975), and regulation of stomatal movement (GO:0010119) and phenylpropanoid metabolic process (GO:0009698) were the top enriched GO terms in the Steelblue module (868 genes) of KRIL8 (**Figure 5D**).

In KRIL37, the Darkmagenta module (988 genes) was enriched in 19 GO terms related to ion transport, cation transmembrane transport, and ATP metabolic processes (GO:0046034) (**Figure 6A**). It included mostly NADH dependent proteins: *Os03g0774200* (similar to NADH–ubiquinone oxidoreductase subunit 8), *Os07g0645400* (similar to NADH dehydrogenase), *OsNIA2* (similar to nitrate reductase), and others. *OsZFP15*, *OsIDD10*, *Os07g0119300* (G2-like TF), *OsHHO3*, and *OsGRAS22* were the key TFs that were highly connected in the module. Improvements in salinity and drought tolerance were observed in *OsZFP15* rice overexpression lines according to Wang and others, indicating its role in stress response (Wang et al., 2022). Response to abiotic stimulus (GO:0009628), photosynthesis (GO:0015979), and chlorophyll biosynthesis process (GO:015995) and other related biological terms were over-represented in the Purple module (367 genes) of KRIL37. The key genes and TFs included *OsNIA1*, OsSTN7, *OsbZIP52*, and *OsNLP3* (**Figure 6B**). The transcript of *OsNLP3* was induced under N starvation and was reported to regulate NUE and grain yield in rice (Zhang et al., 2022). Lastly, the Skyblue3 module (201 genes) of KRIL37 was enriched in GO terms such as response to stress (GO:0006950), plant-type cell wall biogenesis (GO:0009832), cell wall biogenesis (GO:0042546), cellular response to stimulus (GO:0051716), and others (**Figure 6C**). About 10 fascilin-like arabinogalactan proteins (FLAs) highly expressed under low N were grouped in this module. FLAs are suggested to affect the organization of cell wall polysaccharides such as cellulose and pectins, leading to alterations in cell wall properties and impacts on plant growth, including root development (MacMillan et al., 2010; Griffiths et al., 2016). Interestingly, the Skyblue3 module consisted of several calmodulin-binding protein genes (*OsCML10*, *OsCML30*, *OsCML7*, and *IQD1*), which are reported to be involved in calcium-mediated signaling. In *Arabidopsis*, Ca^2+^ was reported to act as a second messenger in the nitrate signaling pathway, implicating it in plant N nutrition (Riveras et al., 2015). *OsLBD3-1* (lateral organ domain protein, *CRL1-LIKE 1*) and *OsMyb61* were the key TF in this module (**Figure 6C**). The TF *OsMyb61* regulates cellulose synthesis in rice and promotes nitrogen utilization and biomass production, making it a key NUE-related protein (Gao et al., 2020).

### 3.6 Relationship between the modules based on enrichment analysis

**Figure 7:**
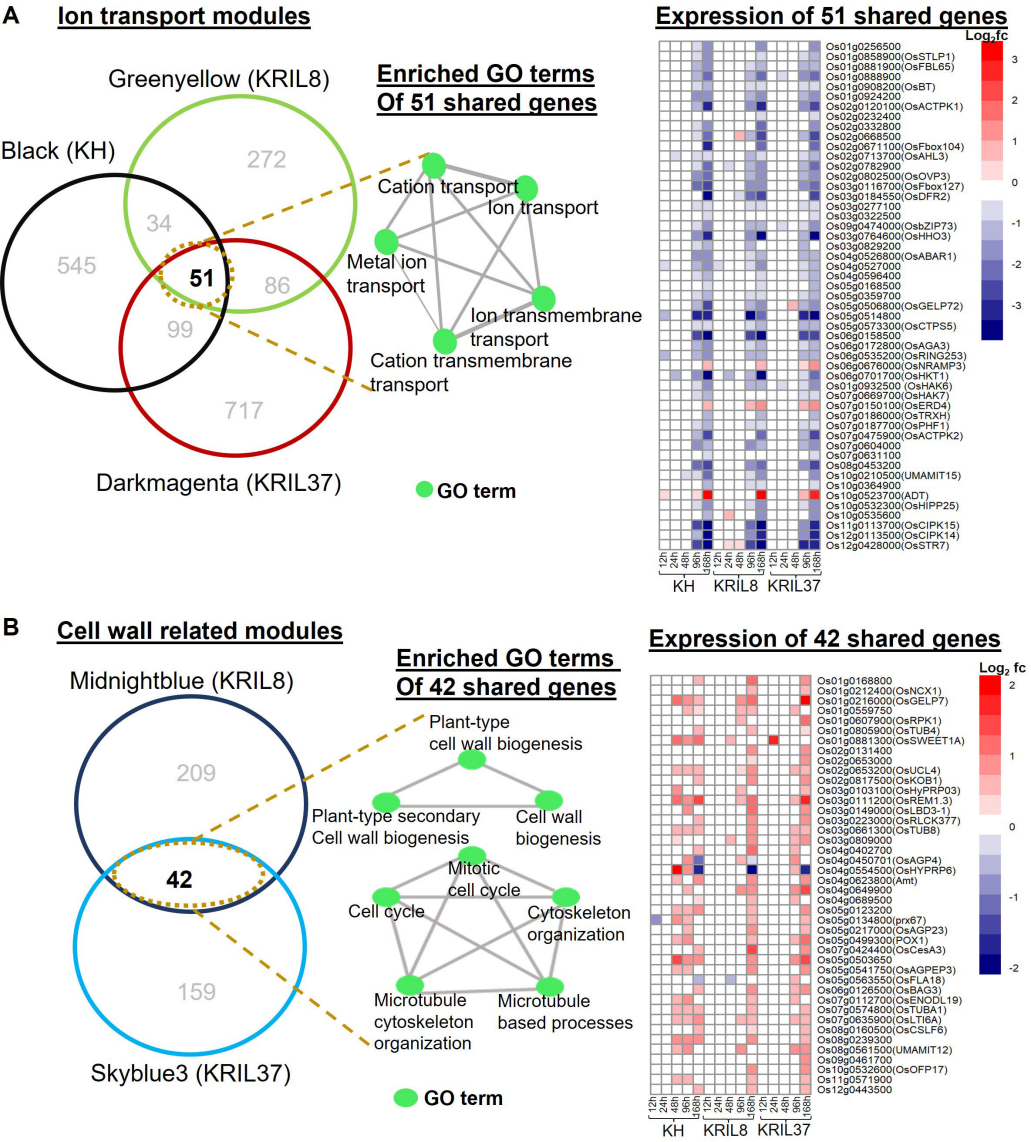
Relationship between significant modules with similar GO terms. **(A)** Venn diagram showing the relationship between the Black module of KH, Greenyellow of KRIL8, and Darkmagenta of KRIL37 involved in ion transmembrane transport processes. The 51 genes shared between the three modules are shown in the intersection. A network of enriched biological terms of the 51 shared genes is shown as well as a heatmap showing the expression pattern of the 51 shared genes under low nitrogen (LN) in comparison to normal nitrogen (NN) in each genotype across the five time points. Most of the genes were downregulated under low N stress. **(B)** Venn diagram showing the relationship between the Midnightblue module of KRIL8 and Skyblue3 module of KRIL37 involved in cell wall organization processes. The 42 genes shared between the two modules are shown in the intersection. A network of enriched biological terms of the 42 shared genes are shown as well as a heatmap showing the expression pattern of the 42 shared genes in each line. Most of the genes were upregulated under low N stress. GO analysis was performed using ShinyGO (v.0.77). The vertical bar indicates relative expression ratio in log2 FC [DEGs were selected at FDR (p ≤ 0.05), and log2 FC ≥ |1| for upregulated and downregulated genes].

Following enrichment analysis of the module genes, the Black module of KH, Greenyellow of KRIL8, and Darkmagenta of KRIL37 were significantly associated with ion transport GO terms (**Figures 4A**, **5B**, and **6A**). Further analysis revealed that these three modules shared 51 genes, which were mainly involved in metal ion transport (GO:0030001), cation transmembrane transport, and ion transmembrane transport and were significantly downregulated as a result of low N stress in all genotypes (**Figure 7A**). They included genes such as *OsHHO3*, *OsACTPK1*, *OsFBOX104* (MAIFI), *OsbZIP73*, *OsBT* (member of the Bric-a-Brac/Tramtrack/Broad family), and several K transporters—*OsHKT1* (high-affinity K^+^ transporter 1), *OsHAK6* (high-affinity potassium transporter 6), and *OsHAK7* (high-affinity potassium transporter 7). This suggests that low N stress suppresses nutrient ion transport across the membrane, particularly potassium ions, as a potential adaptation strategy to facilitate NH_4_^+^ uptake as observed in all the lines. In rice, *OsBT* and *OsHHO3* were reported to negatively regulate N uptake and NUE (Araus et al., 2016; Liu et al., 2023).

In addition to this, regulated cell wall modification through cell expansion and organ growth (particularly root) could be an additional adaptation strategy employed by the introgression lines (KRIL8 and KRIL37) to enhance N uptake under low N stress. The Midnightblue module of KRIL8 (**Figure 5C**) and Skyblue3 module of KRIL37 (**Figure 6C**) shared over 20% of their genes. These shared genes were significantly expressed especially toward the peak of low N stress (96–168 h) and were associated with mitotic cell cycle (GO:0000278), cell wall biogenesis, plant-type secondary cell wall biogenesis, and other related biological processes (**Figure 7B**). Here, low N stress increased the expression of cell wall and cellulose related genes, confirming earlier reports that cell wall biogenesis is associated with carbon metabolism and must coordinate with nitrogen metabolism (Gao et al., 2020). It consisted of cell wall remodeling-related genes such as tubulins (OsTUBs), arabinogalactan proteins (*OsAGP23*, *OsAGP18*, and *OSAGPEP3*), cellulase related genes (*CESA3*, *CslF6*), and *OsLBD3-1* (a lateral organ domain protein, *CRL1-LIKE 1*) (**Figure 7B**).

## 4 Discussion

### 4.1 The ILs maintained high phenotypic performance at the seedling stage

Nitrogen (N) is an essential macronutrient substantially required for plant function and growth. In weight basis, N makes up more than 2% of the plant dry weight and functionally serves as signaling molecule in vital cellular processes like metabolism, storage, and photosynthesis (Tegeder and Masclaux-Daubresse, 2018). However, considering field production and environmental cost, there is a need to improve N use efficiency (NUE) in crops to ensure high productivity under reduced N input to safeguard the environment. Therefore, our understanding of N response at both physiological and molecular levels will facilitate the development of N use efficient cultivars. Physiological analysis showed that KRIL8 and KRIL37 produced bigger shoots under LN than KH. Consistent with previous results, the IL (KRIL37) was found to exhibit longer roots and higher root biomass compared to KH (Adu et al., 2022). In the case of highly mobile nutrients such as N, longer roots are preferred under limited conditions for nutrient foraging (Ogawa et al., 2014). The ILs had bigger tissue dry matter and accumulated more N into their tissues than KH under both N conditions, making them N use efficient (**Figure 1**). N use efficient cultivars have the capacity to accumulate N at the initial growth stage (vegetative) for future remobilization to support growth and development (Nasielski et al., 2019).

### 4.2 Low N stress induces several secondary metabolites

The nitrogen status of the plants plays a significant role in many aspects of a plant’s life, including growth and development and yield, and impacts the accumulation of secondary metabolites. DEGs obtained at the peak (96–168 h) of low N stress were enriched in oxidative stress as a result of accumulation of reactive oxygen species (ROS) and secondary metabolite terms. The production of ROS under low N suggests that the stress is perceived by plants similar to other abiotic stress, including drought and salinity (Schützendübel and Polle, 2002; Sadak et al., 2019). Secondary metabolites such as phenolic acids, flavonoids, terpenoids, steroids, alkaloids, and their derivatives provide antimicrobial and antioxidant (mitigators of oxidative stress) activities although their accumulation is highly dependent on environmental factors such as salinity, light, and nutrient status (Schmelz et al., 2014). Therefore, the elevation of secondary metabolites processes such as diterpenoid, phenylpropanoid, and flavonoid biosynthesis could provide an antioxidant defense system in response to excess ROS produced as a result of low N stress (Khan et al., 2023). In *Artemisia argyi*, low N stress promoted the expression of genes involved in flavonoid synthesis (Wang et al., 2023), indicating that modulating the nitrogen status could potentially alter the plant chemistry. In addition, the nitric oxide biosynthetic process specifically induced in the shoot of KRIL37 could provide extra defense against cell damage as a result of oxidative stress.

### 4.3 Differential expressions of N and C metabolism genes could influence stress adaptation

There was variable expression of several DEGs related to N uptake and assimilation in all the lines used, which could contribute to the differential response of the lines to N treatments. N uptake genes *OsAMT1;3*, *OsAMT2;1*, *OsNRT1.5A*, and *OsNRT2.4* were induced under low N in the roots of the ILs (KRIL8 and KRIL37) not in KH toward the last sampling point. The high-affinity NH_4_^+^ transporters include the *OsAMT1;3* and *OsAMT2;1* with a significant role in NH_4_^+^ uptake under low N conditions. The increase in expression levels of *OsAMT1;2* in the rice root facilitated the higher N uptake, resulting in improved plant growth and nitrogen use efficiency (Lee et al., 2020). Peptide transporters have an important role in organic nitrogen translocation for plants, and this is accomplished by the OPTs (the oligopeptide transporters) and the PTRs (the peptide transporters). *OsPTR7* was non-responsive in the shoot of KH but upregulated in the ILs. *OsPTR7* is a plasma membrane-localized transporter that mediates the uptake of di- and tripeptides into root cells and plays a role in the long-distance transport of organic nitrogen (Komarova et al., 2008). *OsGLR1.1*, *OsGLR1.3*, and *OsGLR3.5* were highly expressed under low N in the shoot of the ILs compared to KH. The role of glutamate receptors in nutrient metabolism (Ca, N) and root morphology is well documented. In nitrogen-starved rice seedlings, glutamate was the most abundant free amino acid and could be easily metabolized and converted to the other nitrogen-containing compounds in rice (Kan et al., 2017; Li et al., 2019). Plants store carbohydrates mostly as starch, which is synthesized in the plastid compartment of the chloroplast in photosynthetic cells. The biochemical pathway of starch biosynthesis in leaves has been well characterized, and it is quite similar to glycogen biosynthetic processes in storage tissues (Nakamura, 2002; James et al., 2003). Starch and cellulose metabolism genes such as *OsESV1* and those annotated as plant glycogenin-like starch initiation protein (PGSIP), including *OsPGSIP-A2*, *OsPGSIP-A3*, and *OsPGSIP-B2*, were significantly downregulated or non-responsive in the shoot of KH in contrast to that of the ILs (**Figure 2B**). PGSIP1 is involved in starch biosynthesis, and knockout lines resulted in reduced starch production in the leaves (Chatterjee et al., 2005). The differential induction of starch-related genes in the ILs in response to N treatment could be a strategy to adjust the nitrogen and carbon balance. In general, the differential expression of these N and C metabolism genes in the lines could be one of the factors contributing to the high NUE in the ILs.

### 4.4 Suppression of ion transport genes could influence low N stress adaptation

Considering the GO analysis, the Black module of KH, Greenyellow modules of KRIL8, and Darkmagenta of KRIL37 shared several genes which were mainly enriched in ion transmembrane transport biological processes, indicating that they could be important modules for N stress response through nutrient uptake and assimilation. The genes involved generally showed reduced expression (**Figure 7A**) in the root under low N, indicating that the suppression of ion transport related genes could be important for the N stress tolerance. Efficient N uptake by plants is critical for achieving NUE (Masclaux-Daubresse et al., 2010); hence, the downregulation of genes that negatively regulate N uptake such as *OsHHO3*, *OsBT*, and *OsACTPK1* could be necessary for enhanced N uptake to combat low N stress. In rice, *OsHHO3* was reported as a transcriptional repressor of ammonium transporter genes, including *OsAMT1;1* and *OsAMT1;2*, such that the inactivation of *OsHHO3* enhanced NH_4_^+^ uptake and improved NUE, indicating its role in the negative regulation of NH_4_^+^ under N deficiency (Liu et al., 2023). The overexpression of *OsBT*, a member of the Bric-a-Brac/Tramtrack/Broad gene family, was reported to negatively affect primary root growth and lower plant biomass in *Arabidopsis* and rice under low nitrate conditions, thereby reducing their NUE (Araus et al., 2016). Similarly, *OsACTPK1* has been demonstrated to suppress HATS (high ammonium transporters) in rice root under sufficient ammonium conditions (Beier et al., 2018); therefore, the suppression of *OsACTPK1* could enhance N uptake. *OsHHO3*, *OsBT*, and *OsACTPK1* were downregulated in all the roots of KH, KRIL8, and KRIL37, indicating that the down-modulation of these genes could be necessary to facilitate N uptake under deficient conditions. Within the ion transmembrane transport related genes, the *OsbZIP73* and *OsHHO3* TFs co-expressed with these genes in all the three genotypes. Liu et al. (2018) reported on the role of the *OsbZIP73* in cold stress tolerance at the seedling and reproductive stage of rice while *OsHHO3* negatively regulated NH_4_^+^ uptake via the repression of a number of N transporter genes (Liu et al., 2023), suggesting their role in abiotic stress response. Therefore, in addition to ammonium transporters, *OsHHO3* could be involved in the transcriptional regulation of other ion transport related genes.

The ion transport module related genes included potassium ion transporters such as *OsHKT1*, *OsHAK6*, and *OsHAK7* that are involved in the primary K^+^ absorption and transport. NH_4_^+^ and K^+^ possess a number of similarities regarding charge, size, and characteristics that are important for their transport across membrane; therefore, the uptake and accumulation of one is shown to influence the other. It is suggested that the transport of NH_4_^+^ and K^+^ across the cell plasma membrane could be subjected to antagonistic interaction; hence, high external K^+^ concentration has been reported to significantly reduce NH_4_^+^ uptake into root cells of barley and rice (Szczerba et al., 2008; Balkos et al., 2010). Therefore, the downregulation of genes involved in ion transport, specifically potassium, could reduce K^+^ transport across the membrane under low N as an adaptation strategy to facilitate NH_4_^+^ uptake.

### 4.5 Cell and cell wall organization could be associated with root growth under low N stress

Contrary to the repression of ion transmembrane transport related genes, 42 genes shared by the Skyblue3 module of KRIL37 and Midnightblue of KRIL8 were significantly upregulated in the root under low N. These genes were mainly involved in cell wall biogenesis and modification biological processes and consisted of cell wall and cellulose proteins such as tubulins (*OsTUB4*, *OsTUB8*, *OsTubA1*), arabinogalactan proteins (*OsAGP23*, *OsAGP18*, *OSAGPEP3*, *OsFLA18*), cellulose synthases (*OsCESA3*, *OsCslF6*), hydroxyproline-rich glycoproteins proline-rich proteins (*OsHyPRP03*, *OsHyPRP06*) and *OsLBD3-1* (*OsCrll1*, *CRL1-LIKE 1*), and others related to tissue growth. According to Gao and others, about 70% of plant photosynthetic products are used to generate cell wall polymers to help build plants; therefore, cell wall biogenesis is associated with carbon metabolism and must coordinate with nitrogen metabolism (Gao et al., 2020). Cell wall modifications play critical roles in plant physiology, not limited to differentiation, plant pathogen interactions, abiotic stress response, plant organ growth, and nutrient acquisition (Fernandes et al., 2013; Marowa et al., 2016).

Cellulose synthase (*CESA*) is a critical catalytic subunit of the cellulose synthase complex responsible for glucan chain elongation (Crowell et al., 2009; Eisinger et al., 2012). Under low N, Rivai et al. (2021) reported an increased expression of *CESA* and *CSLF*, resulting in an enhanced synthesis of glucans and mixed-linkage glucan (MLG). Further analysis revealed that the cell walls of the sorghum seedlings grown under low N were thicker than those of control plants due to the enhanced formation of secondary cell walls resulting in an increase in the amounts of hemicellulose (Rivai et al., 2021). Similarly, arabinogalactan proteins (AGPs) including FLAs (FASCICLIN-LIKE AGPs) are ubiquitous in the cell wall and extracellular exudates and are involved in the function of the cell wall (Showalter, 2001; Ellis et al., 2010). AGPs are reported to be involved in the regulation of plant growth and development, through their role in cell wall structure, and architecture to promote root growth, differentiation, and response to biotic and abiotic stress factors (Nguema-Ona et al., 2012; Tucker et al., 2018). This clearly shows that N deficiency affects the development and remodeling of the cell wall structure and constituents for tissue growth.

The root system of rice is mainly made of post-embryonic stem-derived roots named crown roots (CR) for nutrient acquisition (Coudert et al., 2010), and their development is regulated by several auxin and LATERAL ORGAN BOUNDARIES (LOB) domain proteins. According to Inukai et al. (2005), the *CROWN ROOTLESS1* (*CRL1*) is expressed in parenchyma cells close to the peripheral vascular cylinder of the stem, which is the site for CR initiation. The comparison of *crl1* mutant and wild-type (WT) plants revealed three *CRL1* dependent genes, namely, *FSM* (*FLATENNED SHOOT MERISTEM*)/*FAS1* (*FASCIATA1*), *GTE4* (*GENERAL TRANSCRIPTION FACTOR GROUP E4*), and *MAP* (*MICROTUBULE-ASSOCIATED PROTEIN*), which are reported to be involved in chromatin remodeling, control of cell division, meristem differentiation, and shoot and root development, directly implicating *CRL1* in cell modifications for root development (Coudert et al., 2011). *CRL1-LIKE 1* (*OsLBD3-1*), a paralogue of the *CRL1* co-expressed with these cell wall related and cellulose metabolism proteins in the ILs; *OsLBD3–1* therefore could be involved in the direct regulation of these genes for increased root growth in KRIL8 and KRIL37 for efficient nutrient uptake. Therefore, in addition to N assimilation and C metabolism genes enhancing N uptake and C metabolism, cellulose production and deposition for cell wall maintenance and tissue growth under N stress could be strongly coordinated in the ILs for bigger root biomass, which in turn enhances N acquisition.

However, between the Midnightblue module of KRIL8 and Skyblue3 of KRIL37, the mechanism of low N stress signaling could vary. In addition to the cell wall related and cellulose metabolism terms, the Midnightblue module of KRIL8 specifically consisted of a signal transduction (GO:0007165) and cell communication (GO:0007154) GO terms made up of several auxin transport and signaling genes (*OsPIN1B*, *OsAUX1*, *OsIAA6*, *OsSAUR21*) and serine/threonine kinases, including those involved in brassinosteroid signaling (*OsBRI1*), which could modulate N signaling transduction in KRIL8. In rice, the *OsPIN1b* gene was reported to be involved in the N signaling pathway, and the mutant (*ospin1b*) plants displayed suppressed root development compared with the WT plants due to ammonium deprivation (Gho et al., 2021). Nitrogen signaling was proposed to have an interaction with auxin activity and carbon production (Zhang et al., 2012; Gho et al., 2021). *CRL1-LIKE* 1 (*OsLBD3-1*) gene is homologous to *Arabidopsis LBD16* and *LBD29* that are directly induced by auxin via *ARF7* and *ARF19* and are necessary for lateral root initiation (Okushima et al., 2007; Lee et al., 2019). This shows that auxin signaling and *OsLBD3–1* may interact to promote root growth. Here, *OsPIN1B*, *OsIAA6*, *OsBRI*, and *OsSAUR21* showed increased expression in the root of KRIL8 but not KH nor KRIL37 with *OsCSLF6* and *OsCESA3* involved in cellulose biosynthesis, suggesting that these auxin transporters could be involved in N assimilation signal transduction and cellulose synthesis for increased tissue N and root biomass in KRIL8. In rice, auxin was reported to be involved in ammonium induced nutritropism in the roots (Yamazaki et al., 2024), directly implicating it in root growth and nutrient signaling.

**Figure 8:**
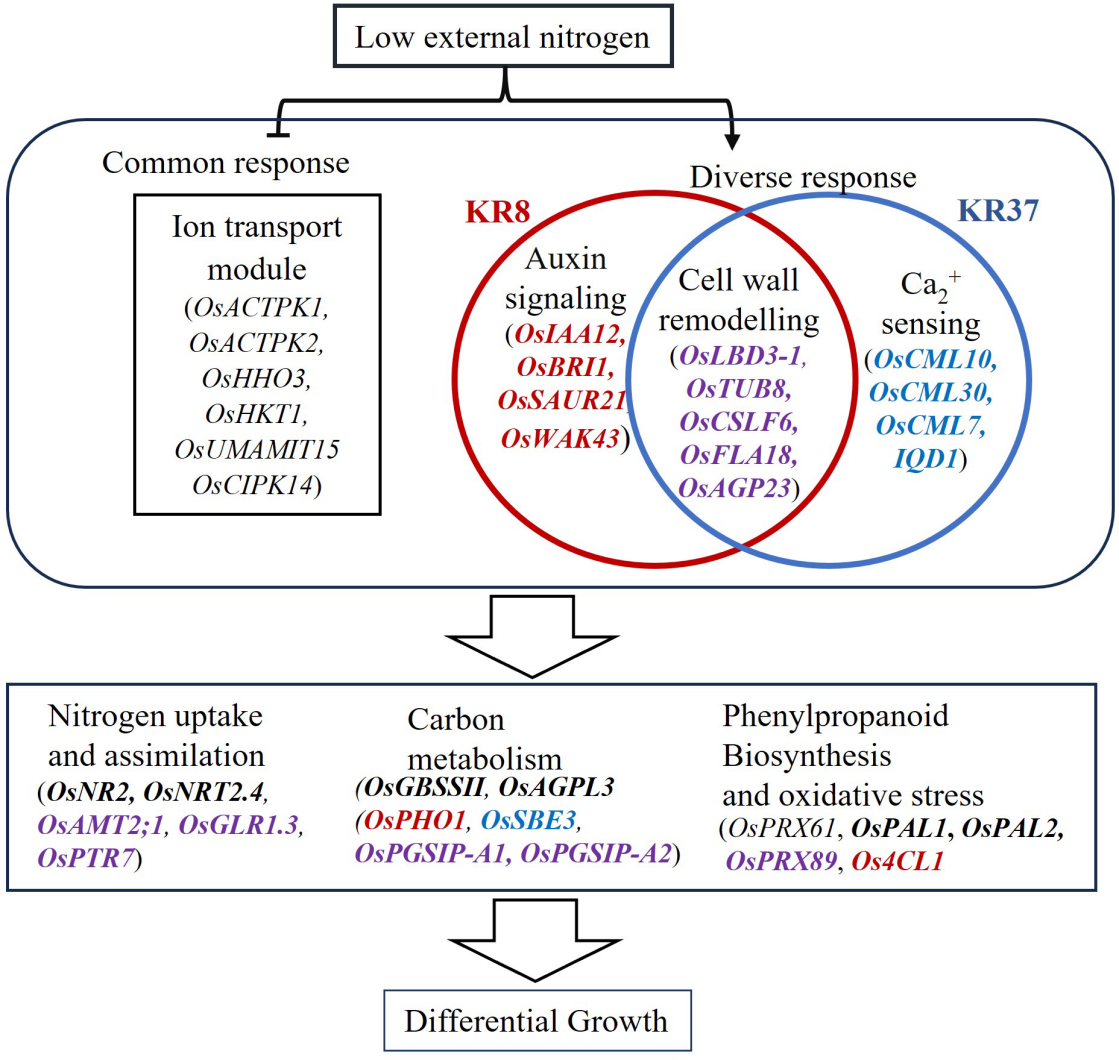
Proposed model depicting the mechanism of low N response in the lines. N deficiency as a signal result in the deactivation of genes involved in the negative regulation of ion transport in all lines. In KRIL8, this signal could be modulated by auxin response genes while that of KRIL37 could be modulated by Ca via calmodulin genes resulting in modification of the cell wall. The downstream regulatory component results in the transcriptional regulation of several pathways involved in nitrogen uptake and metabolism, carbon metabolism, and phenylpropanoid biosynthesis. The unbolded genes are downregulated while those in bold are upregulated. Genes in black are expressed in all the modules of all the lines, while those in purple are expressed in both KRIL8 and KRIL37 modules only. Those in red are specifically expressed in KRIL8 module while those in blue are expressed in KRIL37 module. Arrows indicate positive regulations, and bars indicate negative regulations.

Taken together, *OsHHO3* could be important in regulating ion transport network for enhanced N uptake well as *OsLBD3–1* in modulating cell wall modification through cellulose production and deposition for tissue growth under low N stress for bigger root biomass, which in turn enhances N acquisition and differential growth (**Figure 8**).

## 5 Conclusion

The co-expression analysis revealed that ion transmembrane transport and cell wall organization biological processes could be involved in nitrogen uptake and root tissue development, respectively, under nutrient limiting conditions. KRIL8 and KRIL37 with wild rice genome could employ modification of cell wall components in addition to other pathways as an adaptation strategy toward low N stress. Most of the genes in the cell wall modification related modules may have already been reported in other studies; however, this study shows that these genes may coordinate as shown in the module to adapt to low N stress. The simultaneous regulation of ion transport and cell wall organization genes which is likely coordinated by *OsHHO3* and *OsLBD3-1*, respectively, could influence root development and growth in the ILs (KRIL8 and KRIL37) for enhanced nitrogen uptake under low nutrient conditions.

## Data Availability

The datasets presented in this study can be found in online repositories. The names of the repository/repositories and accession number(s) can be found below: https://www.ddbj.nig.ac.jp/, DRR619887-DRR620084.
