# Supplementary material for: Transcriptome and gene co-expression network analysis revealed a putative regulatory mechanism of low nitrogen response in rice seedlings
Source: Front Plant Sci. 2025 Jun 10;16:1547897. doi: 10.3389/fpls.2025.1547897 (PMC12185425; doi:10.3389/fpls.2025.1547897)

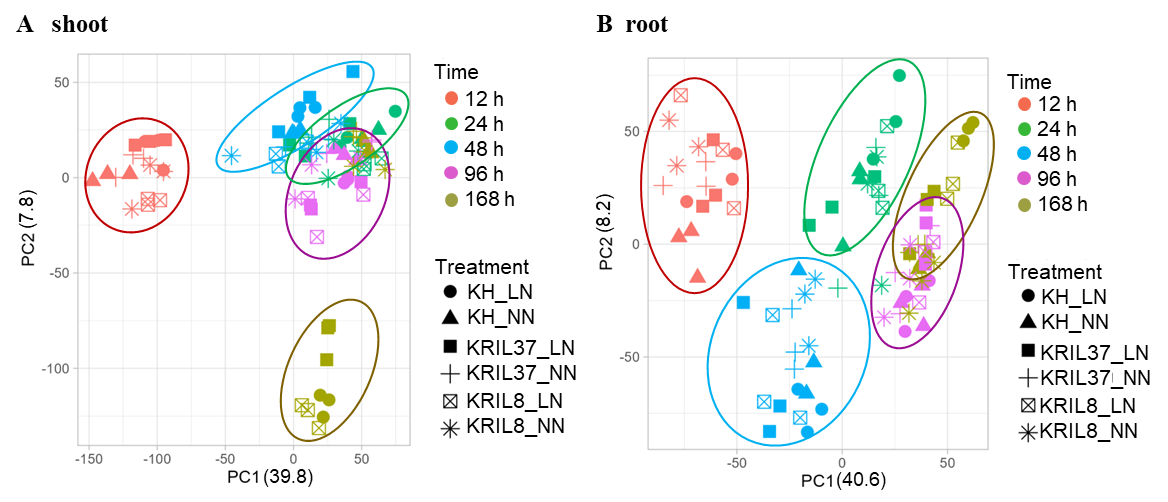


**Supplementary Figure 1. PCA plot of RNAseq shoot and root libraries.**

PCA of count values of RNAseq libraries developed from hydroponic grown seedling (LN, 0.4 mM NH_4_^+^; NN, 1.6 mM NH_4_^+^) for (A) shoot tissues and (B) root tissues.


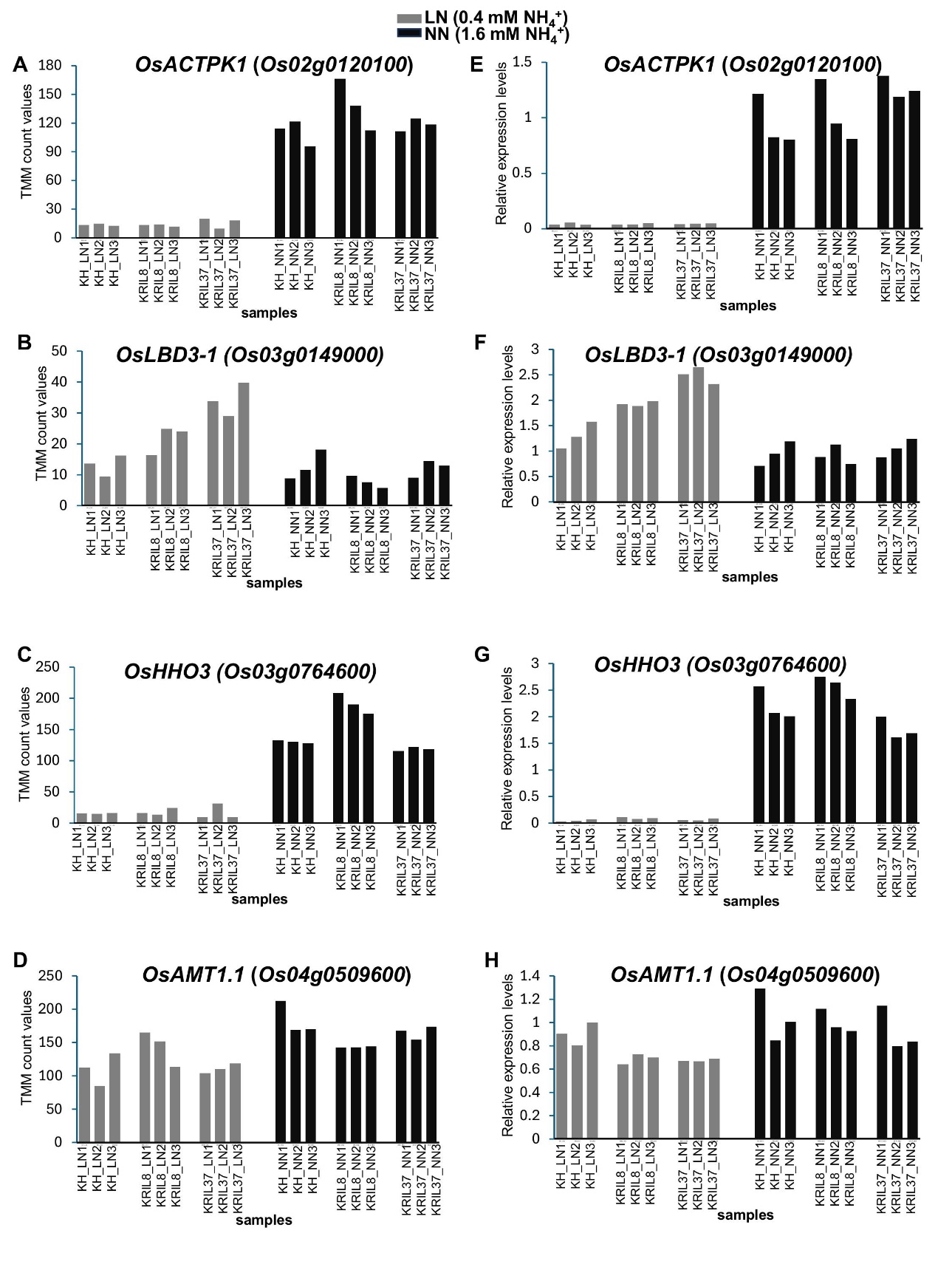


**Supplementary Figure 2**. **Trimmed Mean of M-values (TMM) values and relative levels of four genes**. The TMM normalized values of four genes in the 168hrs samples of the root after sequencing (A-D). The relative expression levels of four genes in the 168hrs samples of the root after qRT-PCR (E-G). Treatments were LN =0.4 mM NH_4_^+^; NN= 1.6 mM NH_4_^+^ while the 1,2,3 indicates biological replicates.

Supplementary Table 1. List of primers used for qRT-PCR.

| **Gene locus ID** | **Symbol** | **Forward (5’-3’)** | **Reverse (5’-3’)** |
| --- | --- | --- | --- |
| Os03g0718100 | *OsACT1* | CTGCGGTATCCATGAGACT | TGGAATGTGCTGAGAGATGC |
| Os02g0120100 | *OsACTPK1* | TTGGCAAGACAAATGGTTCA | ATTGGCCTGGAAATGAGATG |
| Os04g0509600 | *OsAMT1.1* | CGGGTTCGACTACGACTTCT | ATGAGGTAGGCGACGAACTG |
| Os03g0764600 | *OsHHO3* | CCGATTCCGAGAATGACC | GGGTCGTAGAACCACCCTTT |
| Os03g0149000 | *OsLBD3-1* | GAACCCTCTCCTCCAGCATC | TGAGTAGCTGCCTTCCTCCT |

Supplementary Table 2. Enrichment analysis of DEGs (FDR (p ≤ 0.05), and log2FC ≥ |1| ) obtained at each timepoint in KH.


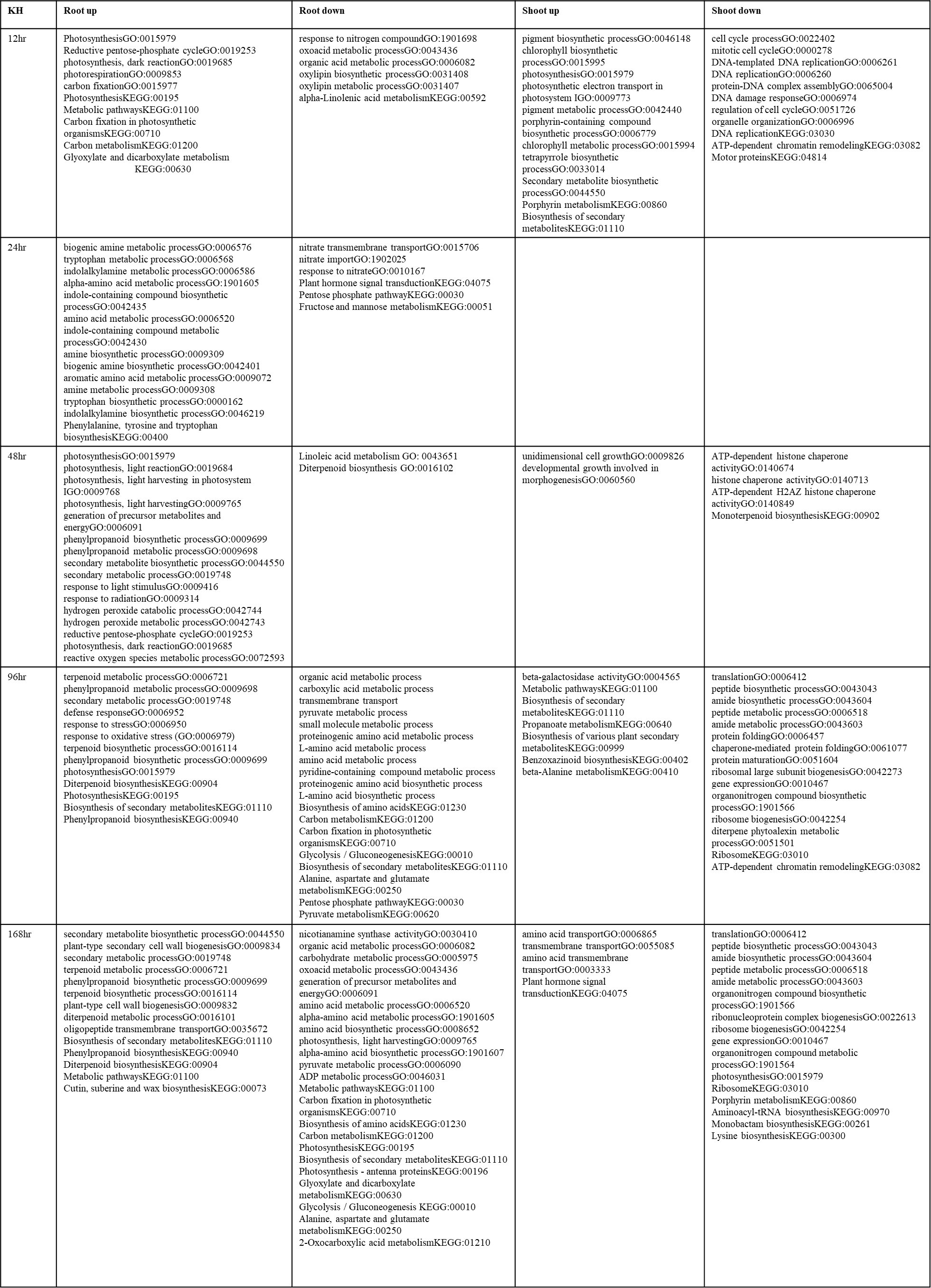


Supplementary Table 3. Enrichment analysis of DEGs (FDR (p ≤ 0.05), and log2FC ≥ |1| ) obtained at each timepoint in KRIL8


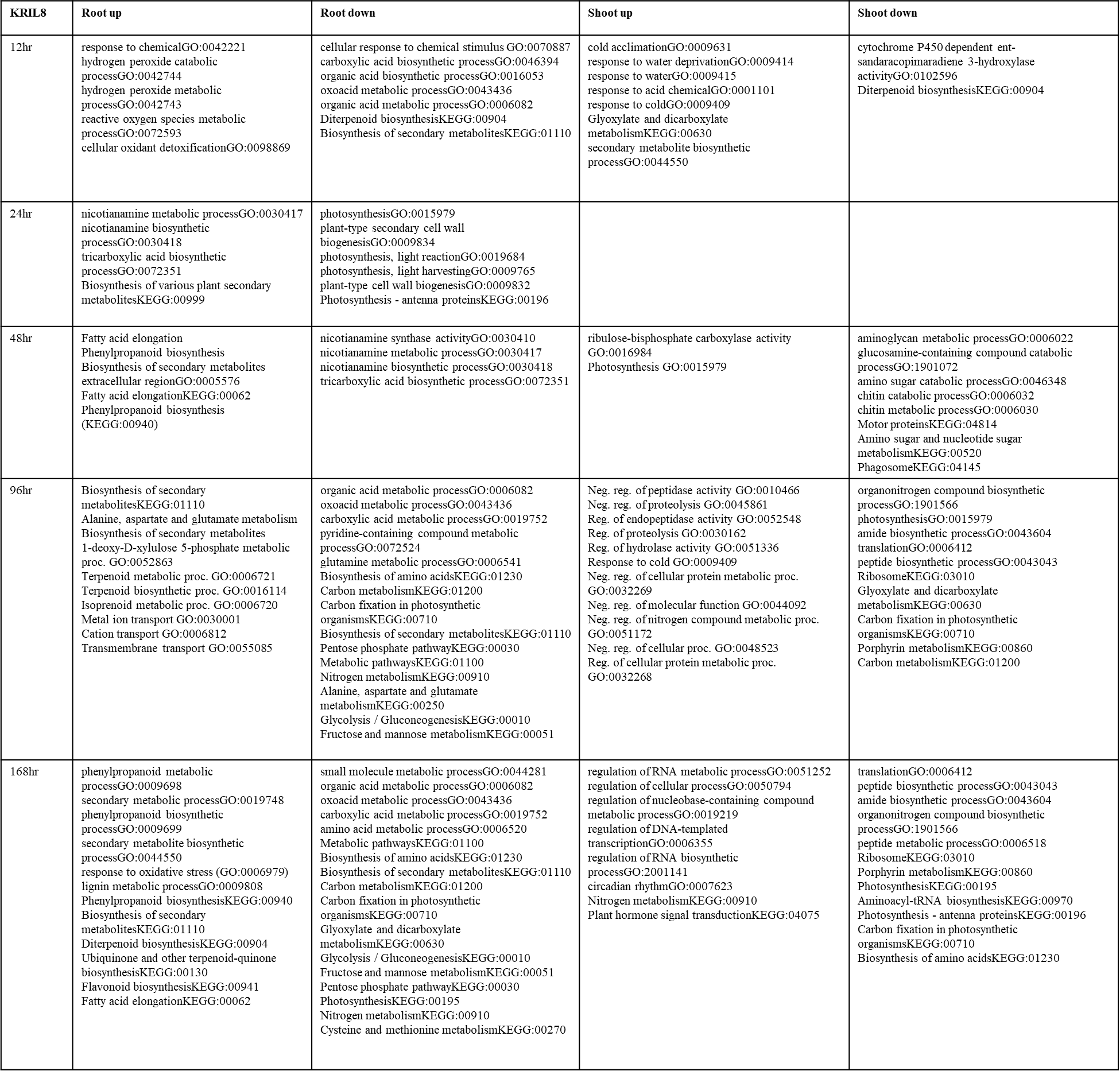


Supplementary Table 4. Enrichment analysis of DEGs (FDR (p ≤ 0.05), and log2FC ≥ |1| ) obtained at each timepoint in KR37


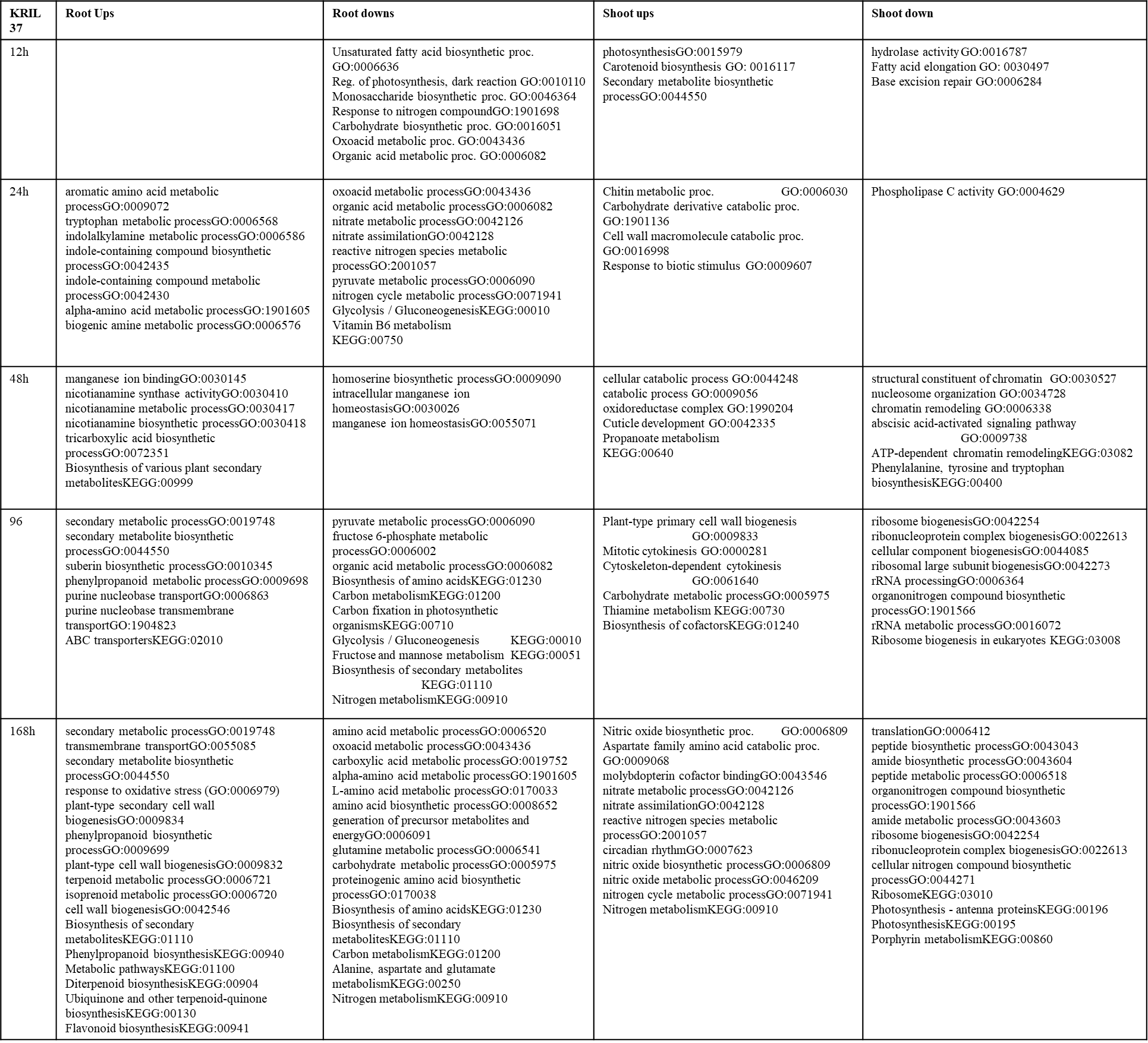

Supplement: Supplementary file 1 [file DataSheet1.docx]
